# Endometrial TGF-β, IL-10, IL-17 and autophagy are dysregulated in women with recurrent implantation failure with chronic endometritis

**DOI:** 10.1186/s12958-018-0444-9

**Published:** 2019-01-03

**Authors:** Wen-juan Wang, Hong Zhang, Zhong-quan Chen, Wei Zhang, Xue-mei Liu, Jiang-ye Fang, Fu-jun Liu, Joanne Kwak-Kim

**Affiliations:** 1 Reproduction Medical Center, Yantai Yuhuangding Hospital of Qingdao University, 20 Yuhuangding East Road, Yantai, 264000 People’s Republic of China; 2 Department of Obstetrics and Gynecology, The Second Affiliated Hospital of Soochow University, 1055th Sanxiang road, Suzhou, 215004 People’s Republic of China; 3 Department of anatomy, Binzhou Medical University, Binhai Road, Yantai, 26400 People’s Republic of China; 4 Central Laboratory, Yantai Yuhuangding Hospital of Qingdao University, 20 Yuhuangding East Road, Yantai, 264000 People’s Republic of China; 5 Reproductive Medicine and Immunology, Department of Obstetrics and Gynecology, Chicago Medical School, Rosalind Franklin University of Medicine and Science, 830 West Court, Suite 400, Vernon Hills, IL 60061 USA

**Keywords:** Repeated implantation failure, TGF-β, IL-10, IL-17, Chronic endometritis, Autophagy

## Abstract

**Background:**

Chronic endometritis (CE) is a condition which results in reduced receptivity of embryos by dysregulated lymphocyte subsets, abnormal expression of cytokines, chemokines and other regulatory molecules in the endometrium (EM). Macroautophagy (autophagy), the highly conserved cellular homeostasis pathway, plays an essential role in the development and function of T lymphocytes, and supports T cell lineage stability and survival fitness. The possible relationships between autophagy and local cytokine milieus in repeated implantation failure (RIF) with CE have not been elucidated yet.

**Methods:**

This case-control study was performed at a large reproductive medicine center between February 2015 and July 2016. Seventy-five recurrent implantation falliure women with CE who had “strawberry aspect” and 75 women with male factor infertility were included. In this study, endometrial expressions of IL-17, IL-10, TGF-β and autophagy related molecules, including LC3-II and mTORC1 were investigated by qRT-PCR, Western blot, immunofluorescence and immunohistochemistry assays.

**Results:**

The expression of IL-17 was significantly higher in patients with CE compared to women with male factor infertility, while the expressions of IL-10 and TGF-β were significantly lower. Moreover, the expression of autophagy (LC3-II) is increased, while the expression of mTORC1 was impaired.

**Conclusions:**

CE is associated with shifted cytokine milieu towards Th17 over Treg immunity in endometrium through impaired autophagy by decreased mTORC1.

## Background

Repeated implantation failure (RIF) is defined as a failure to achieve a clinical pregnancy after the transfer of three or more good-quality embryos in women < 35 years of age, and four or more good quality embryos in women ≥35 years during fresh or frozen embryo transfer cycles [1]. Successful implantation and maintenance of pregnancy require a delicate balance between pro- and anti-inflammatory immune responses at the maternal fetal interface [2]. Endometrial cytokines and chemokines act in complex networks and orchestrate the changes in endometrial leukocyte populations, which play a major role in vascular remodeling and angiogenesis [3, 4]. Chronic endometritis (CE) however, often induces altered endometrial cytokine and chemokine productions, and endometrial dysfunction [5]. In addition, these changes accompany abnormal patterns of lymphocyte subsets and altered secretion of paracrine factors. Hence, CE is often associated with reduced endometrial receptivity to invading embryos and recurrent pregnancy losses [6–8].

In women with CE which is a chronic inflammatory condition, endometrial immune responses are often shifted towards pro-inflammatory profiles and consequently, become unfavorable to invading embryos [9]. Previously, decreased expression of transforming growth factor-β (TGF-β) and interleukin (IL)-10 mainly secreted by T helper (Th) 2, T regulatory cells (Tregs) or alternatively activated macrophage (M2), and increased expression of IL-17 by Th17 cells have been reported to participate in maternal immune rejection of the fetus [10–12]. Hence, a possible assault to the fetus by maternal immune responses can be largely prevented by suppressing Th1 or Th17 immune activation [13–15]. Enhancement of local and systemic Th2 immunity and tolerogenic Tregs can be a possible strategy to suppress the pro-inflammatory immune responses.

The highly conserved cellular homeostasis pathway, autophagy, degrades large protein aggregates, removes damaged or extraneous organelles, recycles nutrients, and promotes cell survival during stressful conditions [16–18]. Autophagy plays an essential role in the differentiation of T lymphocytes and its function. Autophagy is active in Tregs and supports their lineage stability and survival fitness. Genetic or pharmacological alterations in autophagy impair cell survival rate or its metabolism, thereby affecting tissue homeostasis [19]. Decreased expression of autophagy is associated with poor development of embryo and implantation failure [20]. Moreover, Autophagy is physiologically involved in early normal gestation [21]. Microtubule-associated protein 1A/1B-light chain 3 (LC3), is a soluble protein that is distributed ubiquitously in mammalian tissues. LC3 exists in two forms: a cytosolic form (LC3-I) and a lipid phosphatidylethanolamine-conjugated form (LC3-II) that is inserted into both inner and outer membranes of the growing autophagosome. LC3-II, the phosphatidylethanolamine conjugated product of LC3-I obtained after LC3 activation is currently used as a specific marker for autophagy due to its role in autophagosome genesis [22]. mTOR, a master regulator of cellular metabolism, was reported to have a crucial role in regulating cellular autophagy [23–26]. mTOR integrates signals from the environment to the nucleus for the regulation of cell metabolism, proliferation, survival and autophagy [27]. Indeed, mTOR signaling in placenta was positively correlated with birth weight of the infant [28]. Therefore, a balance between autophagy and mTOR may be crucial for successful pregnancy outcome.

Whether the expression of autophagy and mTOR is associated with cytokine network in human endometrium has not been studied well. Considering the importance of IL-10, IL-17 and TGF-β in maternal-fetal immune tolerance, we aim to analyze the endometrial expression of these cytokines and autophagy related molecules, such as LC3II and mTOR1 in women with RIF and CE.

## Methods

### Study population

All study subjects in this case-control study were registered at the Reproduction Medical Center, Yantai, Yuhuangding Hospital between February 2015 and July 2018. The study was approved by the Research Ethics Committee of Yantai Yuhuangding Hospital and all patients signed an informed consent before the inclusion. Patients who are 30 to 35 years old and failed to achieve a clinical pregnancy after 2 or more fresh or frozen embryo transfer cycles with at least three or more good-quality embryos transferred were included. One or two fresh / frozen embryo transferred each cycle done on day 3 (embryo) or day 5 (blastocyst). Women with abnormal uterine cavity (presence of submucosal fibroids or endometrial polyps), uterine malformations (e.g., uterine septum), abnormal karyotype (patient or the partner), antiphospholipid syndrome, diabetes or overt thyroid disease, recurrent spontaneous abortion, hydrosalpinx, pelvic endometriosis, ovulation disorder, endometrial thickness of < 8 mm on the day of hCG administration, ovarian hyper-stimulation syndrome and polycystic ovarian syndrome were excluded from the study. Total, 530 patients with RIF after IVF were investigated by office hysteroscope and 230 (43.4%) out of the 530 RIF patients received a diagnosis of CE by hysteroscopic evaluation and endometrial biopsy. 75 women with confirmed CE who had “strawberry aspect” throughout the endometrium were sequentially included in the study. Controls were seventy-five women undergoing IVF/ICSI (Intracytoplasmic sperm injection) for male factor infertility, whose endometrium was normal by hysteroscopic evaluation. Age, gravidity, BMI, histories of pelvic infection and smoking were not different between the groups. Women with CE had significantly higher numbers of failed embryo transfer cycles as compared with controls (*P* < 0.05) (Table 1).

**Table 1:** Patients characteristics

characteristics	CE group (*n* = 75)	Control group (*n* = 75)	*P* value
Mean age (years)	32.4 ± 0.3	32.1 ± 0.4	NS
Median body mass index (kg/cm^2^)	23.5 ± 0.4	24.8 ± 0.8	NS
Gravidity	0.5 ± 0.1	0.3 ± 0.1	NS
Infertility history (years)	3.5 ± 0.1	2.3 ± 0.1	NS
Smoking (years)	0	0	NS
Number of failed ET cycles	4.0 ± 0.2	0	(*P* < 0.05)
Number of total embryos transferred	3.2 ± 0.2	0	(*P* < 0.05)
History of bacterial or tuberculous pelvic inflammation (years)	0	0	NS

### Evaluation of CE by hysteroscope

Office hysteroscopy was scheduled during the follicular phase (between cycle day 8 to 12) of the menstrual cycle. The procedure was performed using a rigid hysteroscope consists of a telescope with 3 mm outer diameter and 30 degree fore-oblique lens, and a 3-CCD digital camera system (Karl Storz GmbH & Co. KG, Tuttlingen, Germany). A 50-W Hi-Lux light source and a 15-in. video color TELE PACK X LED monitor were used. The exploration of the uterine cavity dwelled on a panoramic view of the cavity followed by a thorough evaluation of the endometrial mucosa, as described previously [9]. All hysteroscopies were performed by the one investigator.

Clinical diagnosis of CE was based on the demonstration of micropolyps (< 1 mm) that fluctuate in the cavity, and the presence of hyperemic endometrium flushed with a white central point, localized or scattered throughout the cavity, referred as “strawberry aspect” [29–32]. All women with clinical CE by hysteroscope underwent endometrial biopsy using a curette for histological confirmation.

### Hematoxylin-eosin staining for the evaluation of CE

Endometrial samples were fixed in neutral formaldehyde solution and later embedded in paraffin for histological analysis. The micro-sections were stained with hematoxylin and eosin. The histological examinations were performed by the single operator who was unaware of the hysteroscopic findings, and the presence of following features were investigated; superficial stromal edema, increased stromal density, pleomorphic stromal inflammatory infiltrates dominated by lymphocytes and plasma cells.

### Investigation of autophage by immunohistochemistry and immunofluorescence

Antigens were unmasked by microwaving sections in 10 μmol/L citrate buffer with pH 6.0, for 15 min, and immunostaining was undertaken using the Rabbit - enhanced polymer detection system with anti- LC3B (bs-2912R, Beijing Biosynthesis Biotechnology Co. Ltd.; dilution 1:800). Color development was performed using HistostainTM-Plus Kits (IgG /Bio, S-A/HRP, DAB, Beijing Zhongshan Goldenbridge Company, China) as a chromogen. After staining, sections were dehydrated through increasing concentrations of ethanol and xylene.

Immunofluorescence studies were conducted in the tissue sections with 4-μm thickness. Primary antibodies were rabbit anti- LC3B (bs-2912R, Beijing Biosynthesis Biotechnology Co. Ltd.; dilution 1:800). FITC conjugated goat anti-rabbit IgG (H + L) (#A22120, Abbkine, Inc.; dilution 1:1200) was utilized as a secondary antibody.

### Quantitative real-time PCR (qRT-PCR)

Total RNA was extracted from decidua with the Rneasy Mini Kits (QIAGEN, Valencia, CA) according to the manufacturer’s instructions. For qRT-PCR, amplification was performed in an ABI5700 (PE Biosystems, Foster City, CA) using the SYBR Green kit (QuantiTect SYBR Green PCR; QIAGEN). The primers were designed from the target human mRNA sequence using Primer Express software (Applied Biosystems). Each primer was entered into an NCBI BLAST search to ensure that it was specific for the target mRNA transcription. The primer sequences are the following: IL-17 forward 5’-CCG GAC TGT GAT GGT CAA-3′, reverse 5′- CTC ATT GCG GTG GAG ATT-3′; IL-10 forward 5’-GAC TTT AAG GGT TAC CTG GGT TG-3′, reverse 5’-TCA CAT GCG CCT TGA TGT CTG-3′; TGF-β1 forward 5’-CAA TTC CTG GCG ATA CCT CAG, reverse 5’-GCA CAA CTC CGG TGA CAT CAA-3′. The housekeeping gene β-actin primers, forward 5′- ACG TTG CTA TCC AGG CTG TGC TAT-3′, and reverse 5′-TTA ATG TCA CGC ACG ATT TCC CGC-3′ were used with all samples. The primers were synthesized by BioAsia Co. (Shanghai, China). The cycling conditions were 15 s at 95 °C, 45 cycles of 5 s at 95 °C, 20s at 60 °C, 10s at 72 °C, and 15 s at 65 °C. Data were analyzed using the GeneAmp 5700 Sequence Detection System software (version 1.1; Applied Biosystems, Foster City, CA) and were converted into threshold cycle (Ct) values.

### Western blot

Endometrial tissues were isolated and washed in cold phosphate-buffered saline (PBS) in pH 7.4, then the tissues were minced in lysis buffer (7 M urea, 2 M thiourea, 30 mM Tris-pH 8.5) containing 50 mM DTT and protease inhibitors at 4 °C for 20 min by applying gentle pressure. The homogenate was then centrifuged at 12,000 g for 15 min, and the collected supernatants were precipitated by 4-fold ice-cold acetone, stored for 1 h at − 20 °C, and centrifuged at 12,000 g for 20 min at 4 °C. Precipitates were washed with 90% ice-cold acetone, dissolved in lysis solution and protein concentrations were determined by the Bradford assay (Bio-Rad, USA). An equal amount of proteins (50 μg) from each sample were separated by 12% SDS-PAGE and were transferred to polyvinylidene difluoride membranes, blocked with 5% (*w*/*v*) skimmed milk for 1 h at room temperature (RT), then incubated for 1 h with primary antibody at RT with gentle agitation. After washing with 0.5% (*v*/v) Tween-20 in Tris-buffered saline for three times, membranes were incubated for 1 h at RT with HRP-conjugated anti-IgG at a final dilution of 1:5000 in TBST buffer. Immunoreactive complexes were visualized using ECL Western Blotting substrate kit (Rockford, IL, USA). Western blot images were quantified by the densitometric scanning with ImageQuant TL 7.0 software (GE Healthcare, USA).

### Statistical analysis

Data are presented as the means ± SEM. Statistical analyses were performed using GraphPad Prism 5 (GraphPad Software Inc.; LaJolla, CA, USA). The Student’s t test was used to compare differences between the two groups. A *P* value of < 0.05 was considered statistically significant.

## Results

### Hysteroscopic finding of CE and histopathology of endometrium

**Fig. 1:**
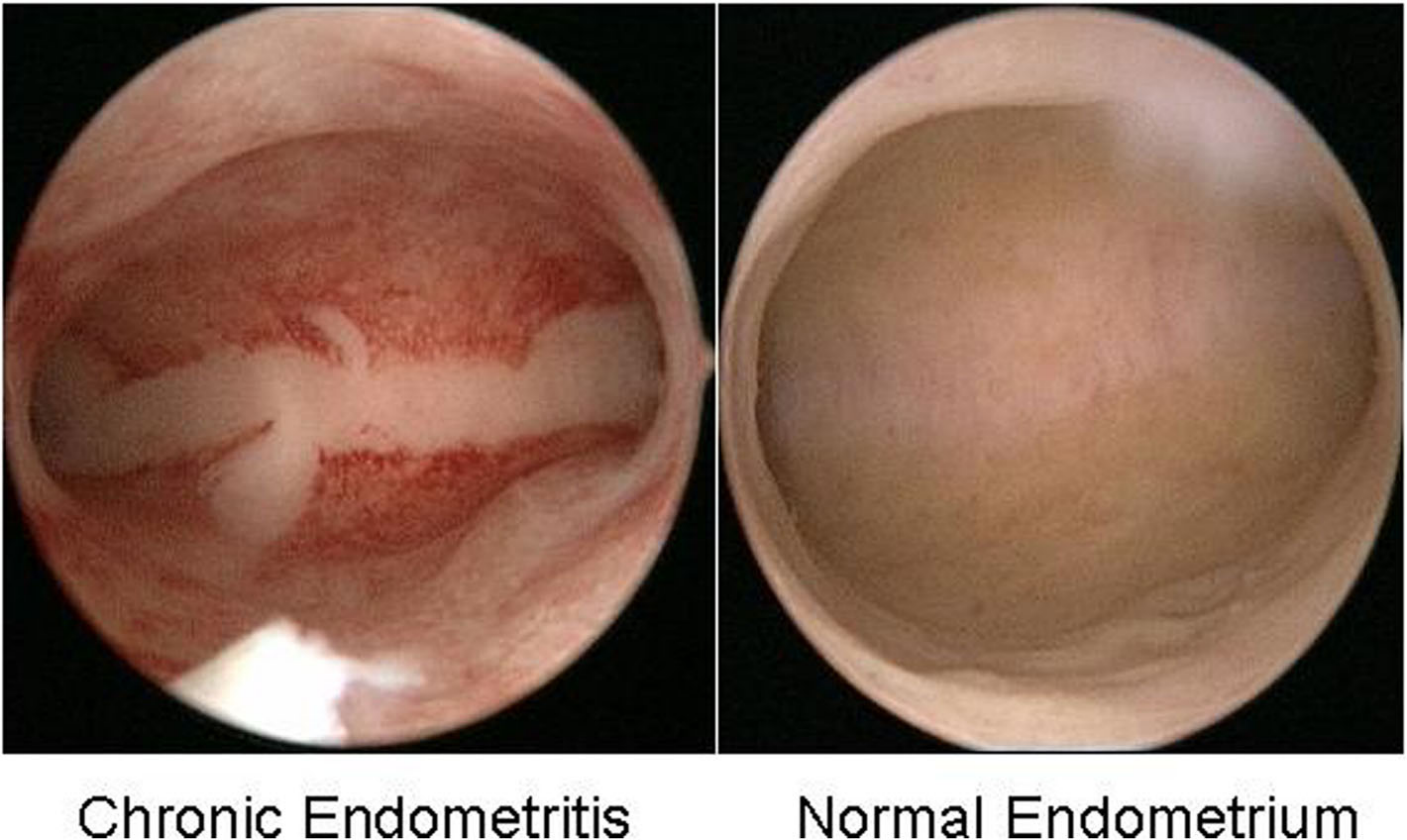
Hysteroscopic finding of chronic endometritis (left) and normal endometrium (right). The focal hyperemic area allowed chronic endometritis to be diagnosed in a woman with a history of repeated implantation failure

The prevalence of CE in women with RIF (*n* = 530) was 43.4% (*n* = 230). In women with CE, focal hyperemic areas, so called “strawberry aspect” was present by hysteroscopic evaluation (Fig. 1).

**Fig. 2:**
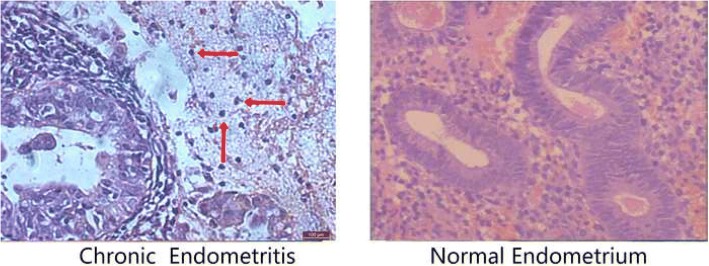
Histological analysis of chronic endometritis (left) and normal endometrium (right). The arrow heads allowed chronic endometritis to be diagnosed in a woman with a history of repeated implantation failure

The histopathological diagnosis of endometritis was determined by HE stain (Fig. 2). With 20x magnification, leukocyte infiltration and changes in the stromal cells were noticed in CE samples. The stromal cells exhibited an increased density, nuclear enlargement, hyperchromasia, and decreased cytoplasm. In a few cases, the stroma resembled fibrous tissues admixed with inflammatory cells. These changes were most often located in the upper half of the mucosa. In the stroma, infiltrates were usually pleomorphic and consisted of lymphocytes admixed with variable numbers of neutrophils, eosinophils and plasma cells. Plasma cells were found in all CE samples. The presence of typical plasma cells in endometrial stromal cells is considered as a diagnosis of endometritis.

### mRNA and protein expression of IL-17, IL-10 and TGF-β in endometrium

**Fig. 3:**
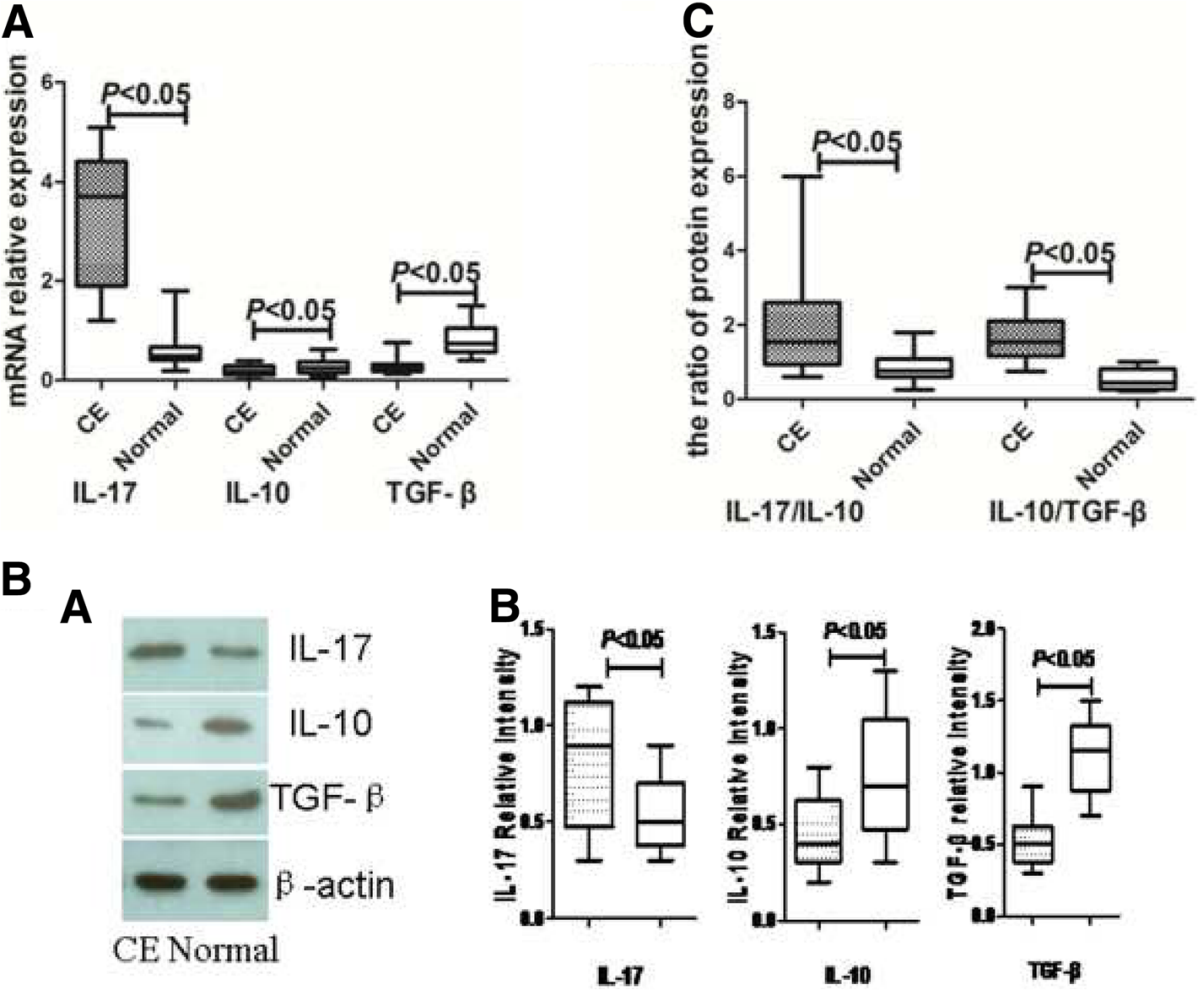
IL-17、IL-10 and TGF-β mRNA and protein expression was determined by qRT-PCR (**a**) and western blotting (**b**) (*n* = 15 for each group). Expression levels were normalized to β-actin

*qRT-PCR* was used to detect the gene expression of related molecules in the endometrium (*N* = 15 for each). Results indicated that IL-17 mRNA expression was significantly higher (*P* < 0.05), while IL-10 and TGF-β1 mRNA expressions were significantly lower (*P* < 0.05 respectively) in the CE groups as compared with those of controls (Fig. 3a).

Western blot analysis was performed to explore the endometrial expression of IL-10, TGF-β and IL-17 (N = 15 for each). The level of IL-17 proteins (0.8 ± 0.1 vs 0.5 ± 0.06) was significantly higher, while the levels of IL-10 (0.5 ± 0.06 vs 0.8 ± 0.11) and TGF-β (0.5 ± 0.05 vs 1.1 ± 0.08) were significantly lower in the CE group than in controls (*P* < 0.05 respectively) (Fig. 3b). Moreover, the ratio of IL-17/IL-10 in the CE group (2.05 ± 0.52) was significantly higher than that of the controls (0.85 ± 0.14) (*P* < 0.05). The ratio of IL-17/TGF-β in the CE group (1.65 ± 0.22) was significantly higher than that of controls (0.54 ± 0.10) (*P* < 0.05) (Fig. 3c).

### The expression of autophagy related proteins, LC3-II and mTORC1 in endometrium

**Fig. 4:**
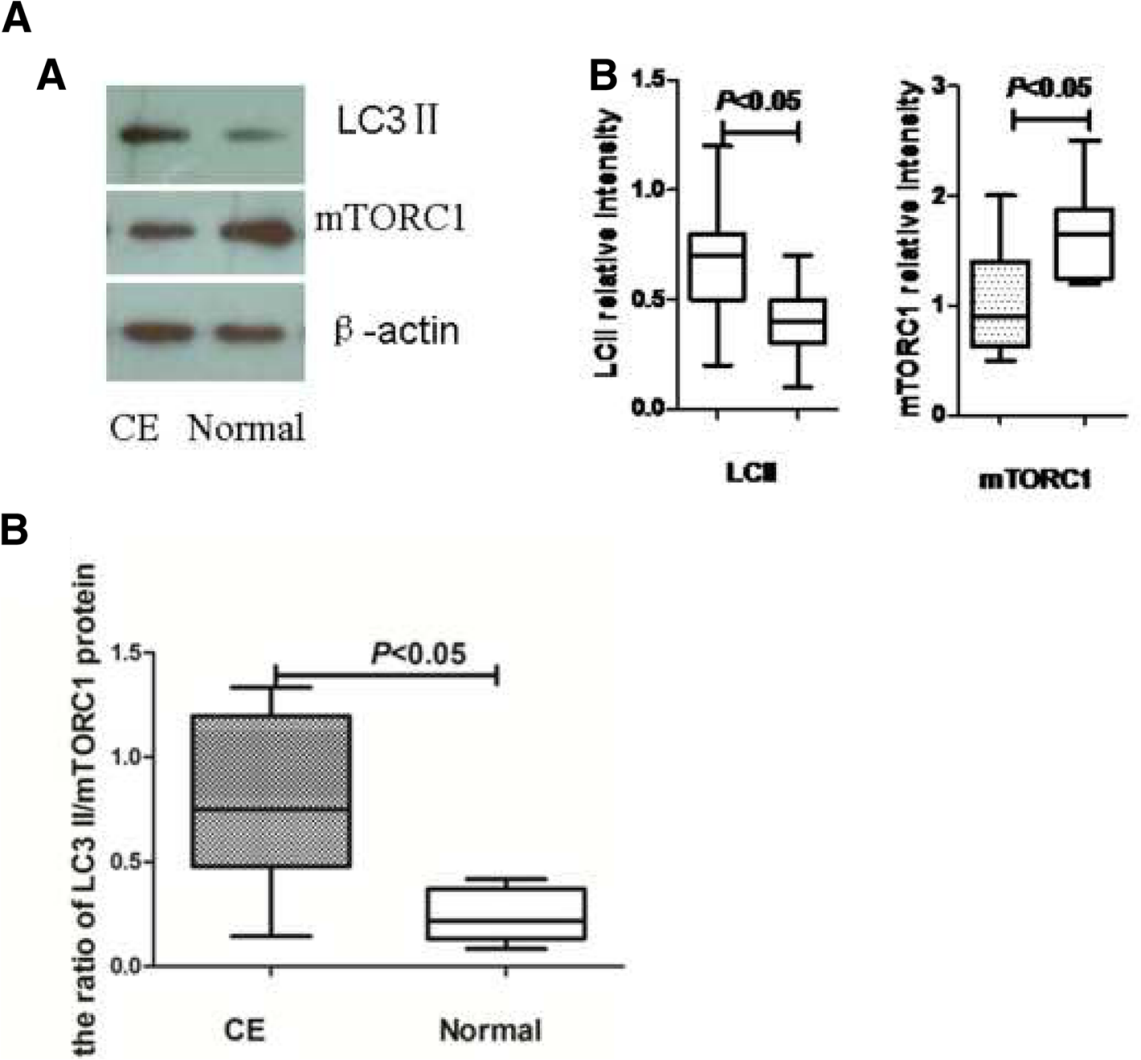
LC3II and mTORC1 protein expression was determined by western blotting (n = 15 for each group). Expression levels were normalized to β-actin

Western blot analysis was performed to explore the LC3-II and mTORC1 (*N* = 15 each). The level of LC3-II protein (0.4 ± 0.05 vs 0.6 ± 0.09) was significantly increased, while the level of mTORC1 (1.7 ± 0.13 vs 1.0 ± 0.15) was significantly lower in the CE group than in controls (*P* < 0.05 respectively) (Fig. 4a). The ratio of *LC3 II/mTORC1* in the CE group (0.78 ± 0.13) was significantly higher than that of controls (0.24 ± 0.38) (*P* < 0.05) (Fig. 4b).

**Fig. 5:**
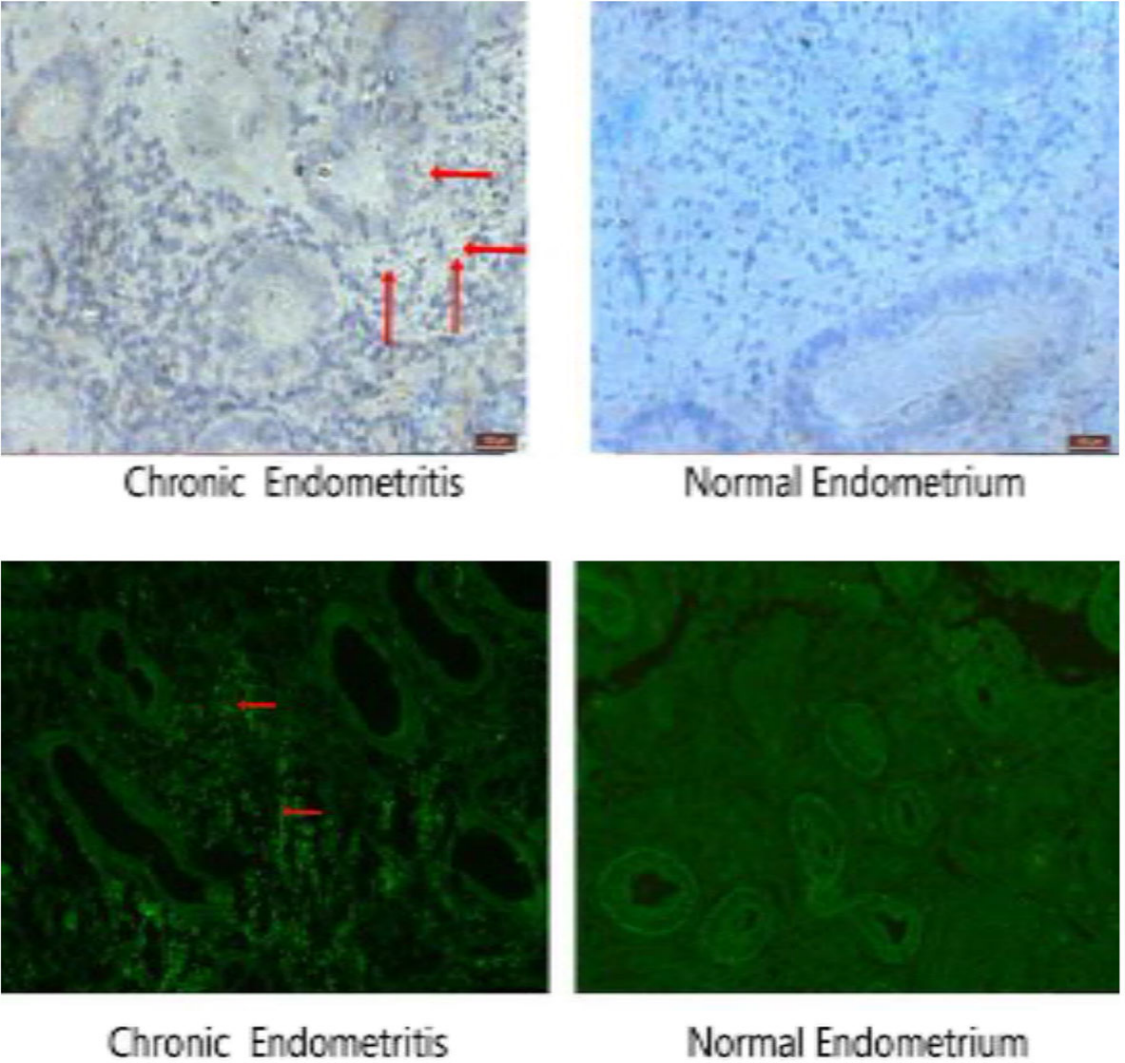
Localization of autophagy in CE and normal endometrium. Localization of autophagy marker (LC3II) by immunohistochemical staining (**a**) and immunofluorescence (**b**) (n = 15 for each group). Signs of autophagy are detectable in endometrial stromal samples but not in endometrial glands

Using immunofluorescence and immunohistochemistry methods, we evaluated the occurrence of LC3-II in endometrial samples. In both CE group and controls, autophagy was clearly detected in endometrial stroma but not in endometrial glands. The expression of LC3-II was significantly higher in endometrial stroma of CE than normal endometrial stroma (Fig. 5a, b).

## Discussion

In this study, we report that women with RIF and CE have decreased endometrial TGF-β and IL-10 expression and increased IL-17 expression, accompanied by increased autophage. These findings support a notion that CE is associated with increased pro-inflammatory immune responses, which is often related to poor reproductive outcome, such as recurrent pregnancy losses (RPL) or RIF [33].

CE is usually asymptomatic and associated with infection, incomplete pregnancy loss or retained placental tissue [34]. Although antibiotic treatment has been frequently recommended, often a causal organism cannot be detected. CE was reported in 14% of women with RPL and 11% of fetal death group. Per-pregnancy live birth rate without the treatment was 7% vs. 56% with treatment [35]. In this study we found 43.4% of women with RIF have CE, which is higher than those of women with RPL or fetal death.

The density and phenotype of lymphocytes in peripheral tissues are generally regulated by local immunoregulatory cytokines, particularly those influencing antigen-presenting cell (APC) function [36], and phenotype acquisition and maintenance in T helper cells [37]. Chronic inflammation may induce an abnormal local immune-regulatory cytokines and alter the pattern of CD4+ T cells. During pregnancy, CD4+ T cells provide a receptive environment for the development of the semi-allogenic conceptus by balancing Th1/Th2 and Th17/Treg cell immune responses [38]. Tregs contribute to the maintenance of tolerance during implantation and suppress maternal alloreactive immune responses against paternal antigens in trophoblasts by actively suppressing self-reactive lymphocytes via mechanisms that are mediated by a cell to cell contact and production of soluble factors, such as TGF-β and IL-10 [10–12]. In this study, women with CE had significantly decreased TGF-β and IL-10 expression in endometrium, which reflect numerical or functional deficiency of Treg cells. Previously, it has been reported that in the absence or the functional deficiency of Tregs, inflammation and fibrosis occur, and blastocysts fail to implant [39, 40]. In our previous study, we demonstrated that decreased expression of Tregs at the maternal-fetal interface in mice with impaired implantation [41]. Contrarily, the periodic accumulation of Tregs during the receptive phase plays an essential role for the proper establishment of pregnancy [42].

Th17 and Tregs present a certain level of plasticity, which has diverse fates in different inflammatory frameworks [43, 44]. Under inflammatory circumstances, Tregs can trans-differentiate into Th17 cells, while Th17 cells that present plasticity towards Th1 have higher survival rate and less senescence than Th1 cells [45, 46]. However, the expression characteristics of Th17 and Treg cells in CE have not been reported. In the present study, we demonstrated significantly increased IL-17 and decreased IL-10 and TGF-β expressions in endometrium of CE patients. This suggests CE induces a propensity to Th17 over Treg immunity in endometrium, which consequently, leads to poor reproductive outcome, such as RIF or RPL.

Autophagy is a highly conserved mechanism of lysosome-mediated protein degradation that plays a key role in maintaining cellular homeostasis by recycling amino acids, reducing the amount of damaged proteins, and regulating protein levels in response to extracellular signals [47]. Autophagy has important effects on the induction and modulation of the inflammatory reaction [48]. In this study, for the first time we detected significantly increased expression of autophagy related protein LC3-II in the endometrium of CE as compared to normal controls. Increased numbers of LC3-containing vesicles and increased LC3 flux indicate active autophagosome formation and clearance [22, 49]. In CE endometrium, autophagy was mainly present in the endometrial stroma where lymphocytes are residing, but not in the endometrial glands. These findings may suggest that autophagy has important effects on the induction of inflammatory reaction in CE and sequential changes in local cytokine milieu. Indeed, excessively stimulated autophage was reported to lead to endothelial cell death that can contribute to plaque destabilization and maintaining inflammatory status of the plaque in patients with atherosclerosis [50].

Autophagy markers are present in cytotrophoblast, syncytiotrophoblast, extravillous trophoblast and decidual stromal cells, and physiologically involved in early gestation. Impaired autophagy at the feta-maternal interface contributes to the pathophysiology of abortion and preeclampsia [27, 51]. The mammalian Target of Rapamycin (mTOR) is an evolutionarily conserved member of the phosphatidylinositol-3-OH-kinase-related kinase (PI3KK) family that plays a central role in the regulation of metabolism, protein synthesis, energy balance, proliferation and survival. mTOR forms the core of two distinct signaling complexes mTORC1 and mTORC2, of which activation are deferentially regulated [52]. mTORC1 can promote lipid biogenesis and energy metabolism through the activation of transcription factors and repress autophagy [53]. In this study, we report for the first time that the expression of mTORC1 was significantly down-regulated in the CE group. It has been reported that mTOR modifies inflammatory responses by modulating immunoproteasomal degradation [54], and inflammatory responses induced by LPS was suppressed by PI3K/AKT/mTOR pathway. Hence, it is postulated that decreased mTORC1 in CE induces increased autophagy and local pro-inflammatory immune responses [53].

In conclusion, the abnormal expression of IL-10, TGF-β and IL-17 in RIF women with CE, combined with decreased level of autophagy demonstrate the presence of endometrial proinflammatory immune responses, which is associated with a decreased endometrial receptivity and pregnancy rate. These results suggested that by regulating autophagy, it may be able to regulate local immune responses and improve the implantation rate in recurrent implantation failure patients with endometritis. Well-designed epidemiological studies are warranted to verify these findings.
